# Engineered Tools to Study Intercellular Communication

**DOI:** 10.1002/advs.202002825

**Published:** 2020-12-21

**Authors:** Benjamin A. Yang, Trisha M. Westerhof, Kaitlyn Sabin, Sofia D. Merajver, Carlos A. Aguilar

**Affiliations:** ^1^ Department of Biomedical Engineering and Biointerfaces Institute 2800 Plymouth Road, North Campus Research Complex Ann Arbor MI A10‐183 USA; ^2^ Department of Internal Medicine Division of Hematology/Oncology and Rogel Cancer Center 1500 East Medical Center Drive, Rogel Cancer Center Ann Arbor MI 7314 USA; ^3^ Program in Cellular and Molecular Biology 2800 Plymouth Road, North Campus Research Complex Ann Arbor MI A10‐183 USA

**Keywords:** biomedical devices, cell–cell communication, high‐throughput sequencing, intercellular communication

## Abstract

All multicellular organisms rely on intercellular communication networks to coordinate physiological functions. As members of a dynamic social network, each cell receives, processes, and redistributes biological information to define and maintain tissue homeostasis. Uncovering the molecular programs underlying these processes is critical for prevention of disease and aging and development of therapeutics. The study of intercellular communication requires techniques that reduce the scale and complexity of in vivo biological networks while resolving the molecular heterogeneity in “omic” layers that contribute to cell state and function. Recent advances in microengineering and high‐throughput genomics offer unprecedented spatiotemporal control over cellular interactions and the ability to study intercellular communication in a high‐throughput and mechanistic manner. Herein, this review discusses how salient engineered approaches and sequencing techniques can be applied to understand collective cell behavior and tissue functions.

## Graphical abstract

Understanding how intercellular communication circuits orchestrate complex biological processes is a fundamental goal of cell biology with therapeutic applications. This review discusses recent advances in microengineered and molecular profiling technologies that offer unprecedented access to the molecular machinery governing cell behavior, and how these tools reveal heterogeneity within networks of interacting cells along spatial and temporal axes.

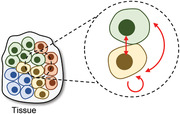

## 1 Introduction

The delicate balance of tissue health and identity is specified through complex and mesoscale communication circuits between cells. Disruptions to intercellular communication from metabolic, mechanical, or biochemical stimuli influence tumorigenesis,^[^ ^1^, ^2^, ^3^ ^]^ metastasis,^[^ ^4^, ^5^ ^]^ aging and senescence,^[^ ^6^, ^7^ ^]^ and autoimmune diseases.^[^ ^8^, ^9^ ^]^ Yet, the precise mechanisms through which individual cells crosstalk and interpret signals from one another to collectively make or maintain tissue‐level decisions remain to be elucidated.

**Figure 1:**
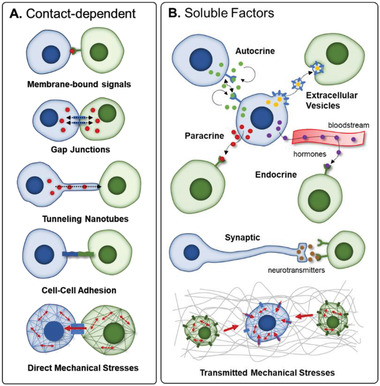
Types of intercellular interactions. A) Effector cells (blue) present signals on their membranes that are recognized by target cells (green), utilize protein structures to shuttle signaling molecules directly between cytoplasms (i.e., gap junctions and TNTs), or form adhesions with homotypic or heterotypic neighboring cells via CAMs. Physically connected cells pull on each other, creating intracellular mechanical tension in the cytoskeleton. B) Secreting cells (blue) release soluble factors that exert range‐dependent effects on specific target cells (green). Additionally, cells stretch and compress the surrounding matrix, exerting mechanical stresses (red arrows) that affect neighboring cells.

Cells within three‐dimensional (3D) tissues communicate through circuits defined by the tissue architecture, whereby the extracellular matrix (ECM), interstitial and vascular flow, and proximity to adjacent cells specify the form of communication. Direct communication occurs through biochemical exchange via cell–cell contacts or mechanical communication through cellular polarization and subsequent tension. Contact‐dependent communication (**Figure** **1**A) is mediated through three methods: 1) mutual binding of cell adhesion markers (CAMs) on interacting cell pairs,^[^ ^10^ ^]^ 2) gap junctions that connect the cytoplasms of neighboring cells,^[^ ^11^ ^]^ or 3) thin membrane projections called tunneling nanotubes (TNTs) that facilitate communication between cells.^[^ ^12^, ^13^, ^14^ ^]^ CAMs encompass a wide variety of transmembrane proteins, including selectins, integrins, cadherins, and members of the immunoglobulin (Ig) superfamily, each with highly cell‐specific functions. Cadherins, for example, permit only homophilic interactions, in which a cadherin on one cell will only bind with an identical molecule on another cell.^[^ ^10^ ^]^ As such, probing these interactions requires techniques capable of deterministically enforcing cell–cell contacts between homotypic and heterotypic cell pairs with temporal resolution.

Indirect cellular communication (Figure 1B) occurs through secreted molecules that are carried by flow or diffusion^[^ ^12^, ^15^, ^16^, ^17^, ^18^ ^]^ from one cell to another, or through large‐scale mechanical tissue deformation and strain in the ECM.^[^ ^19^ ^]^ Soluble signaling is enacted by small, hydrophobic molecules that diffuse passively across plasma membranes, or, more commonly, by hydrophilic molecules that are unable to cross the plasma membrane unaided. Instead, these molecules are recognized by specific receptors on the membranes of target cells and transported into the cytoplasm by pinocytosis, endocytosis, and further directed into compartments by carrier proteins. The mechanism of soluble factor signaling is determined by the nature of the signal and distance between the effector and target cells. Paracrine signals only affect cells in the signaling cell's immediate vicinity, whereas autocrine signals are directed back to the cells that secreted them. Long‐distance communication is mediated by endocrine cells that distribute hormones throughout the body via the bloodstream. Another form of biochemical signaling is through emission of extracellular vesicles (EVs), including exosomes and microvesicles, which permit cells to exchange lipids, proteins, and genetic material.^[^ ^16^, ^17^ ^]^ Besides chemical signaling, cells communicate their state and location by exerting mechanical forces on the surrounding matrix that are perceived by neighboring cells and transduced to direct cytoskeletal rearrangements,^[^ ^20^ ^]^ epigenetic modifications,^[^ ^21^ ^]^ and matrix remodeling programs.^[^ ^22^ ^]^ ECM proteins display nonlinear mechanical properties that amplify applied strains, enabling cells to communicate with neighbors at least five cell diameters away.^[^ ^23^ ^]^ Indirect mechanical communication orchestrates collective cellular behaviors, including migration,^[^ ^24^ ^]^ immune cell recruitment,^[^ ^25^ ^]^ and specialized structures and network formation among endothelial cells.^[^ ^26^ ^]^

Uncovering the molecular mechanisms underlying intercellular communication requires tools that can reconstruct spatiotemporal patterns of cellular architecture as well the biochemical and structural cues found in vivo^[^ ^27^ ^]^ and read outs of the effects of these interactions. In this review, we discuss recent methods for elucidating intercellular communication mechanisms and the effects of these interactions on phenotypic outcomes. We first describe microengineered tools that offer precise spatiotemporal control over interacting cells and can wrap specific microenvironments around the dynamic crosstalk between cells, thereby controlling the microenvironment contexts. Next, we discuss novel high‐throughput strategies that characterize the molecular content of interacting cells at different levels of information. Finally, we provide our integrative perspective on the future of this field.

## 2 Microengineered Approaches to Study Intercellular Communication

**Figure 2:**
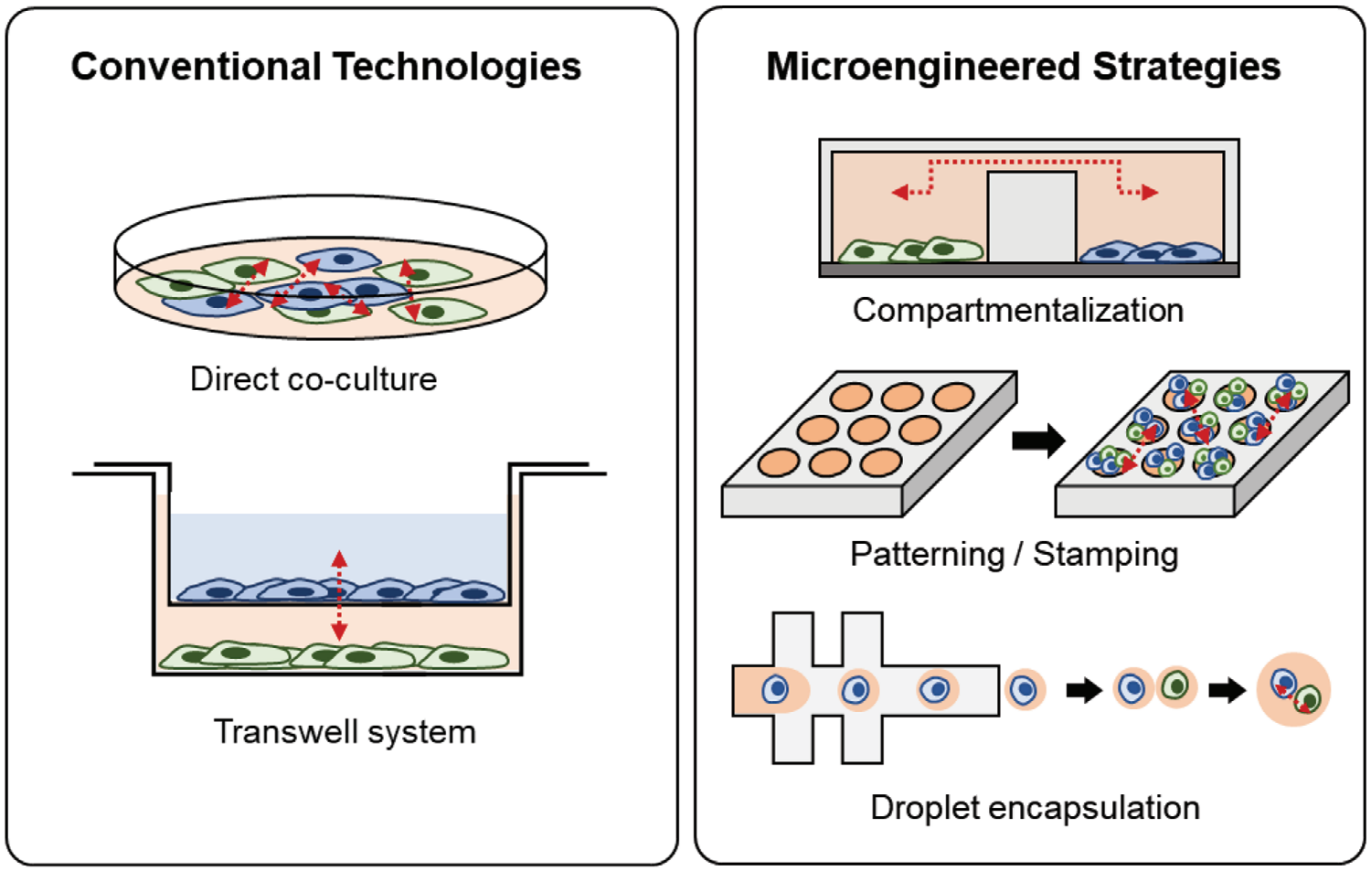
Conventional versus microengineered tools. Direct coculture and transwell systems permit studies of physical and soluble factor signaling but lack spatiotemporal control over cellular interactions. Microengineered tools offer precise control over the local microenvironment and the types of cell–cell contacts through three general strategies: compartmentalization, deterministic cell positioning through patterning and stamping, and droplet encapsulation for single‐cell pairing.

The advent of devices that can match the scale and function of in vivo microenvironments where cells interact, provides a significant advancement over traditional two‐dimensional (2D) cell culture techniques that force cells to grow in planar monolayers^[^ ^28^ ^]^ (**Figure** **2**). These devices are typically fabricated out of polydimethylsiloxane (PDMS), an optically clear, biocompatible polymer that is amenable to cell culture due to its enhanced gas permeability compared to conventional polystyrene substrates.^[^ ^29^ ^]^ Miniature devices designed for cell communication analyses are broadly separated into methods that permit or restrict direct cell–cell contacts.^[^ ^30^ ^]^ The reduction in scale of these devices take advantage of low reagent volume requirements and high‐throughput screening capabilities, making microengineered platforms useful for studying communication in heterotypic and homotypic cocultures.^[^ ^31^, ^32^ ^]^ In this section, we discuss the development of microengineered devices designed to examine direct and indirect intercellular communication.

### 2.1 3D Culture: Organs‐on‐a‐Chip

**Figure 3:**
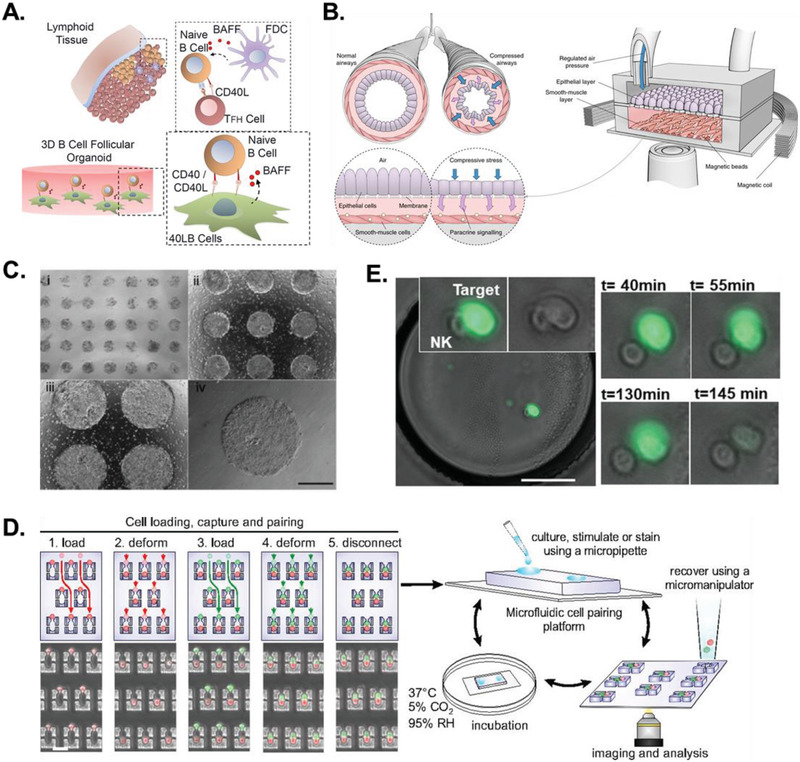
Microengineered strategies to study cell–cell interactions. A) 3D gelatin‐based hydrogel cultures can recapitulate in vivo immune–stromal interactions. Reproduced with permission.^[^ ^38^ ^]^ Copyright 2015, Elsevier. B) Organs‐on‐chips can recapitulate complex physiological responses during homeostasis and disease. Reproduced with permission.^[^ ^47^ ^]^ Copyright 2019, Springer Nature. C) Micropatterning substrates with cell adhesion molecules provides precise control over hESC colony size (i) 200, ii) 400, iii) 800, and iv) 1200 µm in diameter). Scale bar is 500 µm. Reproduced with permission.^[^ ^67^ ^]^ Copyright 2008, Elsevier. D) Microfluidic hydrodynamic traps can pair heterotypic cell types and the consequences of their interactions imaged. Scale bar is 50 µm. Reproducedwith permission.^[^ ^76^ ^]^ Copyright 2016, Proceedings of the National Academy of Sciences (PNAS). E) Heterotypic cells can be coencapsulated in droplet emulsions and their interactions observed in real time. Here, a B cell lymphoma cell line labeled with a viability dye is killed by an unlabeled natural killer (NK) cell over a 145 min time course. Scale bar is 50 µm. Reproduced with permission.^[^ ^84^ ^]^ Copyright 2017, Frontiers.

Cells reside in 3D microenvironments that establish cell–cell and cell–matrix communication networks through structural and biochemical signaling. Elucidating these pathways has long depended on reliable in vitro models that offer cost‐, animal lives‐, and time‐saving benefits compared to animal models, in addition to control over specific culture parameters that affect the dynamics of cellular communication. 3D cell culture systems spatially organize cells in ways that are akin to their native microenvironment, recapitulating cells’ in vivo behaviors and functionality.^[^ ^33^ ^]^ Among an increasing number of 3D culture techniques, 3D hydrogel systems remain one of the most powerful tools to study cell–cell and cell‐ECM interactions.^[^ ^34^ ^]^ Hydrogels are hydrated networks of physically or chemically crosslinked polymers that can be fabricated to encapsulate viable cells, or in the case of 3D hydrogel coculture, multiple types of cells (Ibid). 3D hydrogel coculture strategies are particularly useful when studying deterministic cellular interactions that are sensitive to changes in the surrounding complex cellular architectures. An excellent example of this is the lymph node, which hosts B‐ and T‐cells in spatially distinct regions that meet and facilitate differentiation into short‐lived antibody‐producing plasma cells or form germinal centers (GCs) that produce long‐lived memory B‐cells.^[^ ^35^, ^36^ ^]^ Attempts to model CD4+ (cluster of differentiation 4) T‐cell‐mediated activation of B‐cells through CD40 binding in traditional 2D methods have produced limited quantities of GC‐like B cells that transiently recapitulate in vivo GC dynamics.^[^ ^37^ ^]^ In contrast, a microengineered gelatin‐based 3D hydrogel seeded with engineered stromal cells (**Figure** **3**A) promotes B cell viability ex vivo. The hydrogel is capable of recapitulating the immune–stromal interactions and adhesive ligand patterns present in vivo with sufficient fidelity to yield an ≈100‐fold increase in the production of GC‐like B cells compared to 2D coculture systems.^[^ ^38^ ^]^ By better approximating physiological tissue function, 3D cultures also provide a valuable tool to develop ex vivo disease models for the discovery of personalized therapeutics and the design of novel diagnostics.^[^ ^39^, ^40^ ^]^ However, 3D hydrogels also have limitations. Encapsulating cells in hydrogels is an inherently stochastic process that makes maintaining cells in equal numbers and similar positions across independent cultures difficult, resulting in variability of nonhomogeneous cultures and complicating long‐term analyses. Furthermore, biochemical and genetic analyses are challenged by the need to withdraw encapsulated cells, and the cells are typically exposed to environments that lack the mechanical stresses necessary to promote healthy morphogenesis.^[^ ^41^ ^]^

Organs‐on‐chips can partially overcome the limitations of traditional and hydrogel‐based 3D cultures by integrating 3D culture techniques with microfluidic devices capable of modeling the physical forces found in vivo, such as fluid shear stresses and mechanical compression.^[^ ^42^ ^]^ Using soft lithography techniques, microfluidic chambers can be fabricated in spatial configurations that mimic in vivo tissue architectures to model tissue‐tissue interfaces in ways that 2D and 3D culture do not.^[^ ^43^ ^]^ For example, a lung‐on‐a‐chip mimics air–liquid interfaces of the human lung such as the alveolar–capillary interface by culturing cells on a microporous membrane between chambers of air and blood‐like media that are cyclically stretched to mimic the mechanical process of breathing.^[^ ^44^ ^]^ Integrating mechanical stimulation and fluid flow in a 3D culture environment reconstitutes the respiratory system's physiological response in vitro during homeostasis and disease,^[^ ^45^ ^]^ providing a cost‐effective, clinically relevant disease model.^[^ ^44^, ^46^ ^]^ For example, a microengineered model of the bronchial airways was recently developed to study the molecular mechanisms underlying sustained airway smooth muscle (ASM) contractions in asthma.^[^ ^47^ ^]^ Layers of primary human ASM cells and differentiated normal human bronchial epithelial (NHBE) cells were cocultured in a microfluidic platform that applied air pressure‐driven bronchospasm contractions on the NHBE cells at physiological levels while monitoring the stiffness of ASM cells (Figure 3B). This system uncovered a series of mechanochemical positive and negative feedback loops between ASM and NHBE cells that play out over varying timescales to regulate bronchospasms. Sudden mechanical stresses in the NHBE layer induced the release of spasmogenic factors (ATP, eicosanoids) that rapidly induced stiffening in the ASM layer, while sustained contractile stresses prompted the release of relaxants (PGE_2_, prostaglandin E_2_) that allowed the ASM layer to gradually loosen toward its initial contractile state. These findings were validated in ASM cells from patients with and without asthma, demonstrating this platform's potential in clinical contexts and the value of microengineered models for studying cell–cell interactions in dynamic tissues.

These models are particularly advantageous for drug screening applications where the high cost and low throughput of in vivo assays can be mitigated with low cost microengineered models that can be produced in abundant numbers and recapitulate key cellular interactions in the tissue of interest.^[^ ^48^ ^]^ An additional benefit of microengineered platforms is fine control over indirect tissue‐tissue communication through modulation of 3D soluble factor gradients. For instance, altering pore diameter on PDMS membranes can either permit or exclude certain molecules from participating in intercellular signaling, providing a way to examine systematically the combinatorial effects of secretory determinants.^[^ ^49^ ^]^ Moreover, several 3D culture systems of multiple organs have been assembled together in tandem to monitor organ–organ crosstalk and drug metabolism through their secretory outputs.^[^ ^50^ ^]^ The ability to study tissue‐tissue interactions with precise control is a rich and emerging endeavor to investigate critical tissue‐tissue interfaces across a diverse range of biological systems.^[^ ^42^ ^]^

However, organs‐on‐chips face technical challenges associated with miniaturization. Organs‐on‐chips systems typically culture small cell populations (compared to standard 2D and 3D cultures methods) that make it difficult in some cases to recapitulate the cellular heterogeneity of in vivo tissues and effect the performance of bulk analyses that require large sample sizes. Additionally, while microengineered platforms offer enhanced spatiotemporal control over cell–cell and cell‐ECM interactions, microfluidic devices require more complicated handling than conventional bulk cultures and incorporating physiochemical readouts require advanced in‐house engineering capabilities. Moreover, while these devices can support long‐term cultures (≈1 month)^[^ ^44^ ^]^ that characterize early‐onset diseases, the pathogeneses of many diseases occur over longer periods.

### 2.2 Cell Micropatterning

While extrinsic cues from neighboring cell types and the ECM can be manipulated in compartmentalized microfluidic cultures, such methods typically form randomized cocultures that make studying interactions between multiple cell types challenging. Micropatterned cocultures permit the precise arrangement of heterotypic and homotypic cell types in culture, providing spatiotemporal control over cell–cell contacts. Cell patterns can be formed by parallel laminar flows,^[^ ^51^ ^]^ aqueous two‐phase systems (ATPS),^[^ ^52^ ^]^ or by introducing chemically defined cell capture sites on the cell culture substrate.^[^ ^53^, ^54^ ^]^ Laminar flow patterning and ATPS strategies can produce highly configurable patterns as the boundaries between cells are not defined by solid microstructures. Moreover, since lateral mixing is limited between laminar flows, individual cells can experience chemical gradients when they span multiple streams. However, this property of laminar flows also reduces the effectiveness of studies in heterotypic cell populations, as cellular interactions are limited to adjacent cells. Cell capture sites are commonly defined using microfabrication techniques including microinkjet printing,^[^ ^55^, ^56^ ^]^ microcontact printing (μCP),^[^ ^57^, ^58^, ^59^, ^60^ ^]^ and microstencil patterning (μSP).^[^ ^53^, ^61^, ^62^, ^63^, ^64^ ^]^ A μCP is performed by creating an elastomeric stamp (typically PDMS) of the desired pattern using standard soft lithography techniques that is subsequently coated with “ink” comprised of ECM proteins to facilitate cell adhesion. A μSP is a removable chamber that selectively patterns the substrate with ECM. In both methodologies, the ECM is transferred to a substrate and retains its pattern. Cells selectively adhere onto the patterned molecules for culture. Both μCP and μSP enable the substrate to be patterned with hydrophobic regions to support nonspecific cell adhesion or with antibodies and oligonucleotides that enable hybridization‐based selection of specific cell types.^[^ ^53^, ^65^ ^]^ As micropatterned substrates provide fine control over cell capture sites, cells can be trapped in groups or individually,^[^ ^63^, ^66^ ^]^ exposing population behaviors that are absent in individual cell studies. To pattern cells in different geometries or substrates, multiple μCP or μSP stamps designated for each cell population can be used in series.^[^ ^66^ ^]^

Cells that require microenvironmental regulation in vivo for function have benefitted greatly from micropatterning methods. For example, human embryonic stem cells (hESC) in vivo remain in niche microenvironments that direct their differentiation. Recapitulating control of hESC lineage plasticity in vitro using traditional 2D culture methods is challenging due in part to the heterogeneous responses of hESC populations to environmental cues that produce mixtures of differentiated cells of both endoderm and mesoderm lineages. The μSP technique has been utilized to modulate cell–cell interactions by controlling hESC colony size throughout the differentiation process. Both colony size and stimulation with BMP2 (bone morphogenetic protein 2) and activin A are critical factors that direct hESC fates into extraembryonic endoderm or mesoderm lineage stem cell pools^[^ ^67^ ^]^ (Figure 3C). Micropatterning has also enabled the study of heterotypic cell–cell interactions in complex organs with structurally distinct microenvironments like the liver, where traditional cocultures of hepatocytes with fibroblasts or endothelial cells fail to reconstitute in vivo function. Microstencils were utilized to pattern hepatocytes and a feeder layer of mouse 3T3‐J2 fibroblasts in two distinct configurations. First, hepatocytes were seeded into wells of a microstencil to form monolayers, the microstencil was removed, and fibroblasts were seeded to surround the perimeter of the hepatocyte islands. Second, hepatocytes were permitted to adhere directly on top of a feeder layer of mouse 3T3‐J2 fibroblasts within the microstencil before its removal.^[^ ^64^ ^]^ The microstencil technique that positioned hepatocytes directly on top of the fibroblast feeder layer enhanced hepatocyte function, including albumin production, urea synthesis and glycogen storage.

In addition to cell positioning, micropatterned cell adhesion ligands can be patterned such that the adhesive interactions drive cells to adopt morphologies that induce intra‐ and intercellular stresses.^[^ ^60^ ^]^ Functionalized substrates micropatterned with cadherin and integrin ligands have demonstrated how spatial organization and cross‐talk between cell adhesions influence fundamental cellular behaviors such as proliferation, migration, differentiation, and morphogenesis.^[^ ^68^, ^69^ ^]^ Although μSP is limited by the inherent fragility of PDMS stencils, studies have utilized other more robust materials, such as parylene.^[^ ^70^, ^71^ ^]^ Microinkjet printing methods can be automated by adapting cDNA microarray robots to deposit cells and ECM material in predefined patterns for higher throughput.^[^ ^72^, ^73^ ^]^

### 2.3 Single Cell Pairing

While cell patterning techniques enable the spatiotemporal definition of cellular communities, readouts are still performed in bulk, masking the heterogeneity of intercellular interactions. Elucidating the molecular mechanisms that drive these interactions requires platforms that pair individual cells for subsequent high‐throughput assays. Single‐cell pairing platforms are frequently used to study cellular fusion, a fundamental cellular interaction in which cells merge membranes and exchange cytoplasmic content.^[^ ^74^ ^]^ Hydrodynamic trapping is a common strategy for pairing cells in suspension that flows cells a microfluidic channel and captures them in traps that occupy paths of least resistance.^[^ ^75^, ^76^, ^77^, ^78^ ^]^ Occupied traps increase the local fluid resistance, diverting the remaining cells to unoccupied traps until all traps are filled and the uncaptured cells are washed away. Alternatively, heterotypic cells can be vertically paired in microwells by sequentially centrifuging cell suspensions of each cell type.^[^ ^79^ ^]^ Hydrodynamic methods have been applied to probe interactions in heterogenous contexts such as the immune response, where the number and duration of cell–cell contacts modify immune cell behavior (Figure 3D).^[^ ^76^ ^]^ Pairing natural killer (NK) effector cells with tumor cells uncovered the biological significance of Ca^2+^ signaling in the early stages of NK activation and exposed the heterogeneity of the immune response at the single‐cell level.^[^ ^76^ ^]^

Droplet‐based strategies encapsulate cells in picoliter to nanoliter volumes formed from immiscible aqueous and oil phases to offer higher throughput than platforms that rely on cell trapping within microstructures. Encapsulation rates follow Poisson statistics such that droplet generation flow rates and input cell concentrations can be calculated to maximize the probability of capturing cell singlets or doublets.^[^ ^80^, ^81^ ^]^ Single‐cell droplets from different cell populations can then be paired and fused to form heterotypic cocultures that are treated with varying reagents through fusion with additional droplets.^[^ ^82^ ^]^ Since each droplet acts as an isolated reaction volume, thousands of encapsulated cells and cocultures can be observed over varying timescales in response to user‐specified cell–cell interactions or enzymatic reactions (Figure 3E).^[^ ^83^, ^84^ ^]^ Digital microfluidic (DMF) platforms offer greater spatiotemporal control over encapsulated cells compared to flow‐based droplet generators by replacing pumps and values with electrical, optical, magnetic, or acoustic components that move, mix, merge, and split discrete droplets.^[^ ^85^ ^]^

In summary, microengineered based approaches yield high resolution and deterministic contacts between cells, in pseudophysiological environments. The recent explosion of resources to create or purchase microengineered systems will aid in the adoption of these devices into the biology community and inform our understanding of intercellular communication.

## 3 Spatial Molecular Profiling of Intercellular Communication

Key challenges in studying intercellular communication and spatially dependent traits are representation of the heterogeneity in the molecular identities of interacting cells,^[^ ^86^, ^87^ ^]^ as well as the spatial context of the genomic information. The in vivo niche, in contrast to in vitro settings, consists of spatiotemporally diverse interactions between cells and the surrounding matrix that amplify the intrinsic phenotypic variability between cells, inducing context‐dependent intracellular activity. Thus, isolating the contributions of specific cellular interactions to tissue behavior requires tools capable of profiling molecular content with subcellular resolution, while preserving their spatiotemporal context. Mammalian stem cell niches, for example, comprise microenvironments that provide the soluble factors, extracellular structural components, and cell neighbors necessary to maintain stem cell quiescence and regenerative self‐renewing functions.^[^ ^88^, ^89^, ^90^, ^91^, ^92^ ^]^ Several stem cell niches such as those in hair follicles and intestinal crypts comprise spatially distinct regions that confer varying levels of stem‐like potential.^[^ ^90^, ^93^ ^]^ Thus, mapping gene expression patterns onto spatial coordinates can reveal how specific factors present in vivo govern the determination of stem cell fate.^[^ ^88^ ^]^ Recent advances in single‐cell sequencing (sc‐seq) methods have enabled unbiased, high‐throughput molecular profiling across several layers of biological information (e.g., immunophenotype,^[^ ^94^, ^95^, ^96^ ^]^ epigenetic modifications,^[^ ^97^ ^]^ chromatin accessibility,^[^ ^98^, ^99^ ^]^ transcriptome,^[^ ^100^, ^101^ ^]^ and genome^[^ ^102^ ^]^) that resolve the heterogeneity in cell state and identity within cellular communities.^[^ ^103^ ^]^ However, the dissociation protocols required to produce single‐cell suspensions discard the spatial context of molecular profiles. In this section, we discuss recent developments in sequencing and molecular profiling technologies that retain the subcellular spatiotemporal dynamics of the “omic” layers that define cell state and function.

### 3.1 Direct Sequencing from Intact Tissues

**Figure 4:**
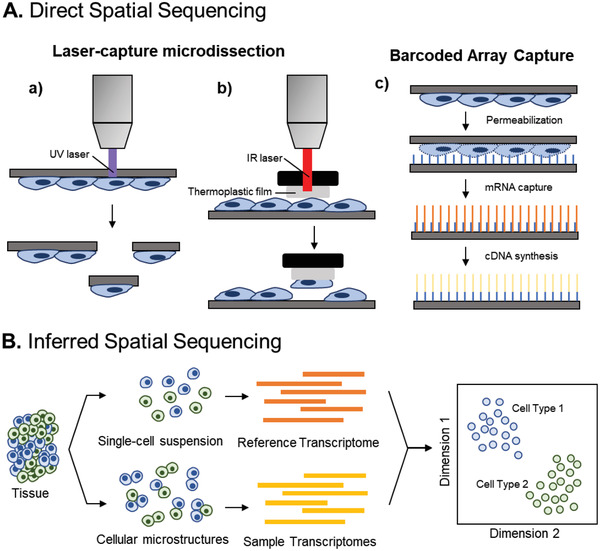
Direct and inferred spatial sequencing. A) Laser‐capture microdissection enables manual selection of individual cells for single‐cell sequencing using a) Ultraviolet (UV) lasers to isolate cells mounted on a photosensitive membrane or b) infrared (IR) lasers to melt a thermoplastic film onto cells to form a polymer–cell composite. Alternatively, cells mounted on glass slides can be fixed, permeabilized, and mounted onto another slide covered DNA‐barcoded beads for mRNA capture and cDNA library synthesis c). B) The cellular composition of manually dissected microstructures can be determined through comparison with panels of landmark genes that have known spatial orientations.

One method of retaining structural and spatial information is to extract molecular material directly from cells in intact tissue sections (**Figure** **4**A). Laser‐capture microdissection (LCM) enables the deterministic capture of targeted cells from stained cryosectioned tissues. After the tissue is imaged, cells of interest are captured by either laser ablation or contact with thermally activated thermoplastic films,^[^ ^104^ ^]^ lysed, and the freed mRNA transcripts prepared into sequencing libraries. By dissecting and collecting cells from serially sectioned tissue slices along an axis, 3D snapshots of gene expression profiles can be generated, thereby charting the spatial relationship between biological function and transcriptional activity.^[^ ^105^, ^106^, ^107^ ^]^

**Figure 5:**
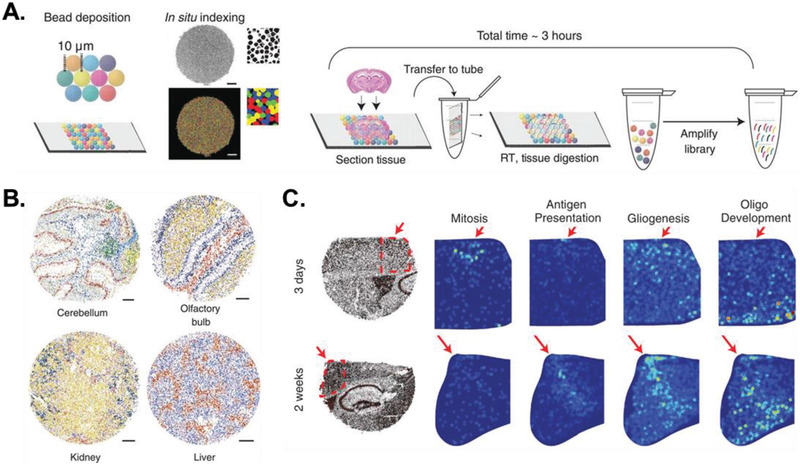
Spatial transcriptomics with Slide‐seq. A) Schematic of how Slide‐seq extracts and reverse transcribes (RT) mRNA transcripts from freshly frozen tissue sections while retaining their spatial context. B) Representative spatial positioning data of individual cells from several tissues colored by cell type. Scale bars are 500 µm. C) Applying Slide‐seq in coronal hippocampal slices of mouse brain tissue at 3 days and 2 weeks after traumatic cortical injury characterizes the transcriptional programs that contribute to tissue regeneration. Gene set enrichment analyses of genes that colocalized with astrocyte and microglia marker genes revealed terms that were enriched at each time point. Red arrows mark the injury site. All scale bars are 500 µm. Reproduced with permission.^[^ ^108^ ^]^ Copyright 2019, American Association for the Advancement of Science (AAAS).

An alternative approach is to capture spatial gene expression patterns across entire tissue sections at once using spatially barcoded RNA‐capture oligonucleotides. Slide‐seq^[^ ^108^ ^]^ (**Figure** **5**A) and high‐definition spatial transcriptomics^[^ ^109^ ^]^ (HDST) operate by depositing fresh‐frozen cryosectioned tissues onto glass slides or coverslips coated with islands of spatially indexed cDNA barcodes that bind mRNA transcripts at resolutions of 10 and 2 µm, respectively, for subsequent RNA‐sequencing (RNA‐seq) library preparation. Spatial coordinates can then be assigned to each RNA‐seq read by matching spatial DNA barcodes on the slide with RNA‐seq library barcodes (Figure 5B). These methods are appealing as low‐cost and rapid (≈3 h) forms of spatial transcriptomics that require minimal expertise in microscopy and sequencing. Moreover, applying these techniques and single‐cell RNA‐sequencing (scRNA‐seq) analysis methods to serially sectioned tissues can resolve spatial variations in both gene expression and cell composition in a tissue volume. This approach can reveal the mechanisms by which individual cell–cell interactions are modulated by their spatial positioning to instruct tissue‐level behavior. For example, performing Slide‐seq in mouse brain tissue sections at several time points following traumatic injury identified spatiotemporally restricted expression of cell type marker genes (e.g., Vim and Gfap in astrocytes) that were spatially correlated with specific biological pathways at each time point^[108]^ (Figure 5C). These analyses exposed the transcriptomic dynamics of astrocytes and microglia as coordinate tissue regeneration at the injury site by upregulating cell cycle genes after 3 days and upregulating genes related to the immune response and restoration of the glial compartment after 2 weeks. Thus, this technique allows researchers to profile not only physical locations of interacting cells, but also the molecular programs that are modulated by such interactions. Combining these temporal studies with serial sectioning would interrogate these processes in three dimensions, revealing how networks of interacting cells evolve in response to biological processes such as development, disease, and aging.

### 3.2 Inferred Cell–Cell Signaling

Cell–cell interactions can be computationally inferred from small cell clusters obtained through manual microdissection or partial enzymatic digestion.^[^ ^86^, ^110^, ^111^ ^]^ Clusters can be submitted for scRNA‐Seq and interacting cell types identified by marker genes or through comparisons with reference transcriptomes (Figure 4B).^[^ ^86^ ^]^ While this approach loses the absolute location of cell types within tissues, it enables the identification of preferential physical interactions in vivo, without requiring prior knowledge of the constituent cell types. In tissues with established spatial landmark genes such as liver lobules, absolute spatial information can be retained by sequencing transcriptomes from cell doublets containing hepatocytes and liver endothelial cells (LECs) and inferring LEC positioning from hepatocyte gene expression.^[^ ^110^, ^112^ ^]^

**Figure 6:**
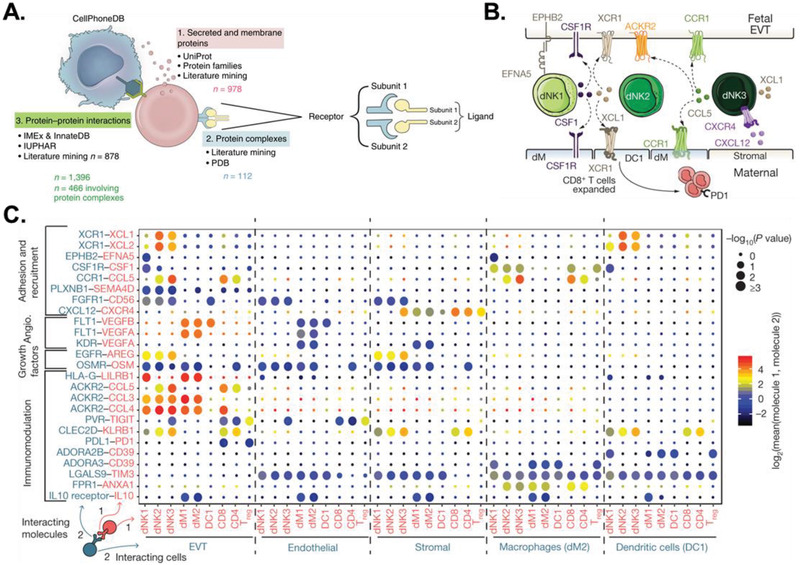
Inferred cell–cell signaling through ligand–receptor interaction analysis in CellPhoneDB. A) Schematic of data sources used to form the ligand–receptor database. Reproduced with permission from CellPhoneDB.org. B) Main ligand–receptor interactions in decidual natural killer (dNK) cell subtypes involved in cell adhesion and cellular recruitment at the maternal–fetal interface. C) Selected ligand–receptor interactions (*y*‐axis) against cell types (*x*‐axis) from a subsampled dataset of human decidua samples. *P* values are indicated by circle size. The means of the average expression level of interacting molecule 1 in cluster 1 and interacting molecule 2 in cluster 2 are indicated by color. Reproduced with permission.^[^ ^118^ ^]^ Copyright 2018, Springer Nature.

Another computational method for predicting cell–cell interactions through scRNA‐Seq is mapping potential ligand–receptor interactions that facilitate physical cellular communication.^[^ ^113^, ^114^, ^115^, ^116^, ^117^ ^]^ Scoring the expression of receptor genes and their cognate ligands can reveal putative interactions that are enriched between different cell subpopulations, identifying soluble factors and cell surface marker signaling pathways that contribute to tissue behavior in homeostasis and disease.^[^ ^115^ ^]^ This approach is particularly powerful for uncovering novel ligand–receptor interactions and inferring their impact on the transcriptional networks of interacting cells.^[^ ^116^ ^]^ In the early stages of pregnancy, for example, predicted ligand–receptor interactions in scRNA‐seq datasets of human decidual tissues using the CellPhoneDB database (**Figure** **6**A) identified cell–cell signaling pathways that prevent maternal immune cells (e.g., decidual natural killer cells, dNKs) at the maternal–fetal interface from targeting burgeoning trophoblast cells^[^ ^113^, ^118^ ^]^ (Figure 6B). While transcriptomic analyses segmented dNKs into three main subsets (dNK1, dNK2, and dNK3) based on the expression of shared and unique marker genes, ligand–receptor analyses in CellPhoneDB identified potentially unique inhibitory cell–cell communication mechanisms between each subset and placental extravillious trophoblasts (Figure 6C). These results highlight intercellular interactions that are key to successful pregnancies and may be perturbed in diseases that affect the early stages of gestation. As these methods depend on computational modeling, inferred interactions must be experimentally validated through in vivo or in vitro studies. Identifying colocalized ligand–receptor pairs and cell‐specific markers in histological sections can provide spatial context to inferred signaling networks and determine whether the signaling molecules facilitate contact‐dependent or independent communication. Alternatively, perturbation experiments can verify cell–cell communication through specific ligands and receptors by inhibiting their function at genetic or post‐translational levels. Overall, these methods enable systematic screening of ligand–receptor interactions within cell populations to predict novel cell–cell interactions, the mechanisms by which those interactions are carried out, and their role in regulating cellular behavior.

### 3.3 Imaging‐Based Transcriptomic Profiling

A fundamental technique for characterizing spatiotemporal heterogeneity in gene expression and tissue architecture is imaging RNA transcripts through single‐molecule fluorescence in situ hybridization (smFISH). Fixed cells or tissue sections are incubated with panels of sequence‐specific DNA probes coupled with a single fluorescent probe, enabling the unbiased detection of individual mRNA transcripts.^[^ ^119^, ^120^ ^]^ Targeting cell type marker genes can quantify environmental contributions to transcriptomic variations within cell types. Compared to sc‐seq techniques, smFISH methods possess greater RNA detection efficiency and provide direct visualization of RNA molecules, making them useful for sensitive measurements of spatiotemporal gene expression patterns and the identification of rare transcripts in rare populations of cells.^[^ ^121^ ^]^ Moreover, the sensitivity and accuracy of smFISH methods make them ideal for verifying RNA expression values found using single‐cell sequencing methods.^[^ ^122^ ^]^ However, these methods require expensive probes, substantial sample preparation, reagent optimization steps, and limited throughput (tens to hundreds of unique transcripts). Additionally, single molecules produce low signal intensities that are difficult to resolve in samples with high levels of autofluorescence, inherent probe hybridization errors introduce off‐target effects, and the number of detectable features is restricted to the number of available fluorescence channels.^[^ ^53^, ^123^ ^]^

**Figure 7:**
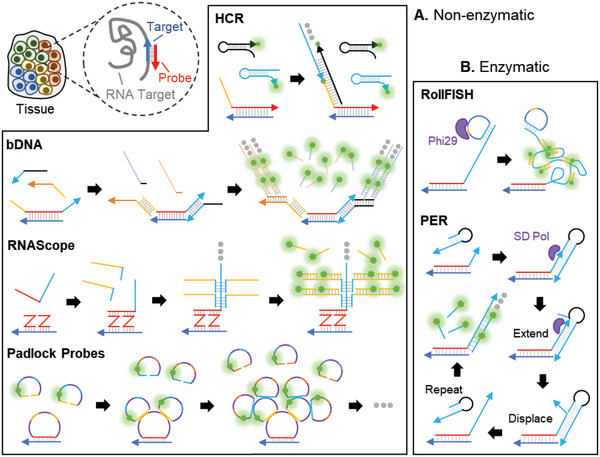
Enzymatic and nonenzymatic methods for amplification of FISH signals. A) Nonenzymatic methods rely on hybridization and strand displacement to enlarge the binding substrate available to fluorescent probes (green stars). Following binding of the primary probe (red) to the target mRNA or cDNA region (dark blue), additional complementary probes are added for sequence‐specific signal amplification. In HCR, initiator probes attached to the primary probe triggers the self‐assembly of fluorescent semi‐stable hairpins into an oligonucleotide sequence. bDNA and RNAscope involve the sequential hybridization of secondary and tertiary probes that massively increase the number of binding sites for sequence‐specific fluorescent probes. Padlock probes implement a similar strategy using circularized probes instead of linear ones. B) Enzymatic strategies use Phi29 (RollFISH) or strand‐displacing (SD) polymerases (PER) to generate concatenated binding sites for fluorescent probes.

To address these limitations, novel signal amplification methods have been developed that reduce the need for highly sensitive microscopy systems while offering transcriptome‐scale multiplexing capabilities. Recent smFISH methods have implemented various enzymatic and nonenzymatic strategies to increase fluorescence signal‐to‐noise ratios. Nonenzymatic methods such as the isothermal hybridization chain reaction (HCR),^[^ ^124^ ^]^ branched DNA (bDNA) amplification,^[^ ^123^, ^125^ ^]^ z‐probes (RNAscope),^[^ ^125^ ^]^ and padlock probe amplification,^[^ ^126^, ^127^ ^]^ assemble large DNA scaffolds on smFISH probes that contain multiple secondary or tertiary probe binding sites that amplify FISH signals (**Figure** **7**A). Enzymatic methods such as rolling circle amplification (RCA)^[^ ^128^ ^]^ and the primer exchange reaction (PER)^[^ ^94^, ^129^ ^]^ amplify existing tags on smFISH probes to provide binding sites for secondary fluorescent probes (Figure 7B).

**Figure 8:**
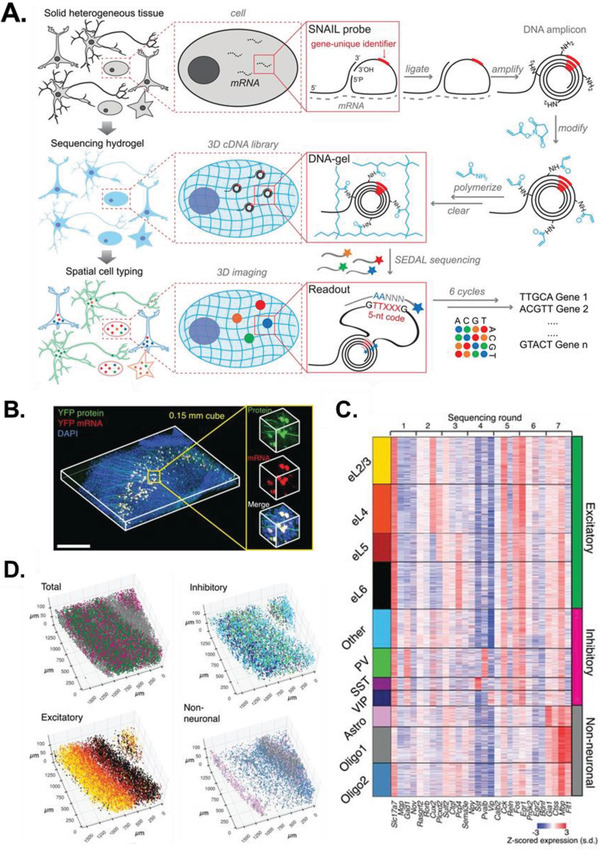
Imaging‐based spatial transcriptomics with STARmap. A) Schematic showing how solid tissues are transformed into cleared DNA–hydrogel structures and dual probe‐labeled genes are read out through sequencing with error reduction by dynamic annealing and ligation (SEDAL). B) Representative image of STARmap applied to 150 µm thick tissue sections comprising YFP‐expressing neurons in the visual cortex of mouse brains. Scale bar is 0.5 mm. C) Heatmap of the normalized per‐cell expression of 28 genes in >30 000 cells from one tissue section grouped by cell type (excitatory, inhibitory, and non‐neuronal) and subtype. D) Spatial distributions of each cell type and subtype. Reproduced with permission.^[^ ^130^ ^]^ Copyright 2018, American Association for the Advancement of Science (AAAS).

A central challenge of enzymatic smFISH protocols is developing protocols that are capable of efficiently navigating dense tissue structures, particularly when the target molecules are short or rare. Spatially resolved transcript amplicon readout mapping (STARmap) bypasses this issue by transforming 3D tissue samples into cubic millimeter volumes of optically transparent hydrogel‐tissue hybrids that preserve biomolecules in their native positions for multiple downstream readouts (**Figure** **8**A).^[^ ^130^, ^131^ ^]^ This approach has been particularly useful for mapping the cellular and transcriptomic complexity of the brain, a highly complex tissue that relies on specific 3D interactions between diverse cell types that are typically only resolved with thick tissue sections.^[^ ^132^ ^]^ A dual oligo approach is implemented to reduce background fluorescent noise that requires both a primer and a padlock probe encoding a gene‐unique identifier to hybridize target mRNA sequences before the padlock probe is amplified by RCA. Amine‐labeled nucleotides are incorporated into the RCA reaction that facilitate the conversion of solid brain tissue into a DNA–hydrogel structure that is cleared of unbound lipids and proteins. The amplified gene‐unique identifiers are then read out in situ using two‐base encoding for error correction (SEDAL), a custom sequencing method that mitigates sequencing errors arising from high fluorescent probe densities. Together, these advances have enabled the simultaneous profiling of 1020 genes from >30 000 cells in 150 µm thick sections of the mouse visual cortex (Figure 8B), revealing new neuronal and non‐neuronal subtypes with unprecedented spatial resolution (Figure 8C,D). Analyzing the distance between individual cells revealed that inhibitory neurons tend to cluster with neurons in their own subtype rather than with other inhibitory subtypes or excitatory neurons and non‐neuronal cells. This suggests that inhibitory neurons form gap junctions with each other to propagate synchronized electrical signals. As STARmap is compatible with in vivo experiments and is not technically limited by the number of mRNA species it can simultaneously profile, it represents a powerful tool for spatial transcriptomics.

Several novel methodologies have also been developed to offer order of magnitude increases in the number of transcriptomic targets per experiment through combinatorial barcoding schemes.^[^ ^94^, ^123^, ^126^, ^133^, ^134^ ^]^ By reiteratively hybridizing, imaging, and removing fluorescent oligonucleotides in predetermined patterns, each RNA species is assigned a unique barcode from a theoretically unlimited pool of sequences.^[^ ^135^ ^]^ This method has been used effectively in sequential fluorescence in situ hybridization (seqFISH+), which employs a palette of 60 “pseudocolors” to profile >10 000 RNA targets in regions of the mouse brain using three fluorescent channels.^[^ ^134^ ^]^ This immense throughput enables smFISH to be implemented in discovery‐oriented studies of spatial gene expression similar to scRNA‐seq.

### 3.4 Multiplexed Spatial Immunophenotyping

Recent estimates from The Human Protein Atlas predict that ≈38% (8500) of human protein‐coding genes encode secreted or membrane‐bound proteins,^[^ ^136^ ^]^ and that these proteins constitute ≈19% (3625) of the human proteome, indicating that at least one out of five proteins in any given cell is involved in cell–cell communication.^[^ ^137^ ^]^ Signals sent or received by these proteins propagate throughout cellular networks and are spatiotemporally regulated to direct cellular and tissue‐level decisions. Spatial proteomic assays can generate a systems‐level understanding of these networks by directly measuring how specific proteins guide cell–cell interactions. Conventional in situ methods such as immunohistochemistry (IHC) enable targeted proteomic profiling and retain spatial context with subcellular resolution but face challenges in target multiplexing and sensitivity due to the spectral overlap of fluorescent tags.

**Figure 9:**
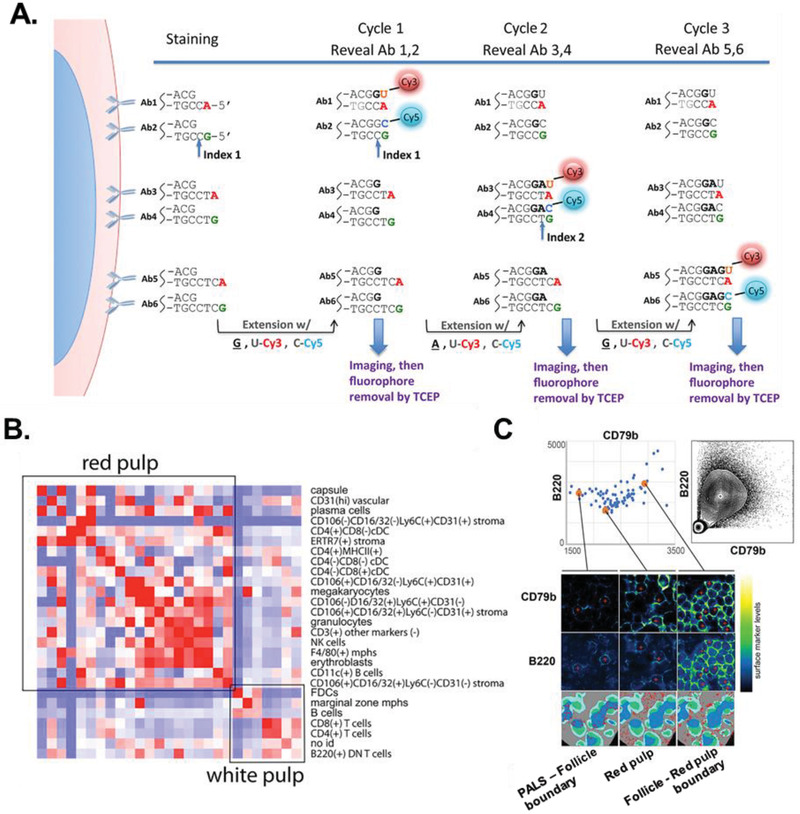
Multiplexed spatial immunophenotyping using CODEX. A) Schematic of barcoding scheme in CODEX. B) Heatmap of the average strength and direction (red, strongly positive; blue, strongly negative) of associations between cell types in normal spleens from a panel of 28 antigens. Large clusters comprising cells in the red or white pulp of the spleen are outlined in black. C) Top left: B220 and CD79b surface marker expression in B cells from normal spleens. Top right: B220 and CD79b surface marker expression from flow cytometry data of isolated splenocytes. Bottom: Levels of B220 and CD79b expression in B cells at different locations of the spleen. Reproduced with permission.^[^ ^133^ ^]^ Copyright 2018, Elsevier.

Methodological principles developed for smFISH techniques have recently been applied to in situ immunophenotyping to achieve greater multiplexing without loss of sensitivity. These methods simultaneously label fixed, permeabilized tissues with multiple DNA‐barcoded antibodies that are read out through cyclic exchanges of secondary fluorescent probes.^[^ ^129^, ^133^, ^138^ ^]^ For example, CO‐Detection by indEXing (CODEX) can simultaneously quantify the expression of up to 66 proteins using in situ polymerases that extend unique antibody‐conjugated oligos in the presence of fluorescent and nonfluorescent nucleotides that confer sequence specificity (**Figure** **9**A).^[^ ^133^ ^]^ This technique not only recovered the spatial distributions and immunophenotypic subtypes of cells in murine spleens, but also generated cell neighborhood graphs that revealed enriched associations or aversions between splenic‐resident cell types. For instance, NK cells clustered were positively associated with F4/80^+^ macrophages while B cells were negative associated with CD4^+^ and CD8^+^ T cells (Figure 9B). Moreover, while the majority of positive and negative associations during homeostasis were homotypic (e.g., B cells exhibited enriched interactions with other B cells), interacting cell pairs were frequently separated and paired with different cell types at varying stages of lupus, an autoimmune disease (e.g., B cells exhibited enriched interactions with CD4^−^/CD8^+^ dendritic cells in the early stages of lupus). Placing the surface marker signatures of cell types in their spatial context further revealed that their immunophenotypes are modulated by the composition of their niche. While B cells in the red pulp of the spleen expressed B220 and CD79b cell surface markers at low and intermediate levels, respectively, their expression levels varied when compared to B cells at the boundary of the red pulp and B cells that resided between follicles and the periarteriolar lymphoid sheath (Figure 9C). Thus, these analyses resolve immunophenotypic heterogeneity and identify specific cell–cell interactions that help maintain tissue homeostasis or become perturbed due to disease.

Protein epitope multiplexing can be improved by conjugating antibodies with transition metal isotopes instead of fluorescent tags and reading out the results with mass spectrometry. This approach, called mass cytometry (commercialized as mass cytometry by time of flight, CyTOF), eliminates spectral overlap and sample autofluorescence to enable the detection of potentially >100 protein targets in millions of cells^[^ ^139^ ^]^ but discards information about tissue architecture.^[^ ^140^ ^]^ Two recent advances in mass cytometry, imaging mass cytometry (IMC, commercialized as Hyperion Imaging System)^[^ ^141^ ^]^ and multiplexed ion beam imaging time of flight (MIBI‐TOF),^[^ ^142^ ^]^ overcome this limitation by quantifying metal isotope content in intact, antibody‐labeled tissue sections. In IMC, labeled tissue sections are ablated spot‐by‐spot with a laser for CyTOF analysis, while MIBI‐TOF rasters tissue sections with a stream of primary oxygen ions that displaces antibody‐bound metal isotopes, as secondary ions are propelled into an orthogonal accelerated TOF mass spectrometer. Metal isotope composition can then be mapped back onto the tissue sections to generate highly multiplexed, spatially resolved images of protein expression. IMC was recently expanded to enable simultaneous detection of both RNA transcripts and protein targets, enabling studies of correlations between gene expression and protein signaling.^[^ ^143^ ^]^ Coupling high‐dimensional mass cytometry and imaging data with computational tools such as histoCAT can quantitatively and systematically identify enriched cell–cell interactions,^[^ ^144^ ^]^ providing a comprehensive analysis of the relationship between cell–cell communication and the surrounding microenvironment.

### 3.5 Live Imaging

Intravital microscopy (IVM) is a powerful tool for directly observing cell–cell interactions in vivo with subcellular resolution.^[^ ^145^ ^]^ Tissues of interest that are optically inaccessible can be surgically exposed for terminal studies^[^ ^146^ ^]^ or covered with transparent windows for repeated imaging.^[^ ^147^, ^148^ ^]^ Scattered fluorescent signals can be resolved with unprecedented clarity by advances in confocal and multiphoton microscopy that reject out‐of‐focus signals outside the focal plane.^[^ ^149^ ^]^ Together, these approaches can reveal the real‐time dynamics of cell–cell interactions in healthy and diseased tissues with recent emphases on the immune^[^ ^150^ ^]^ and invasive tumor microenvironments.^[^ ^151^ ^]^ For example, multiphoton IVM recently revealed that regulatory T cells (Tregs) exert immunosuppressive functions in pancreatic ductal adenocarcinoma through prolonged interactions with intratumoral dendritic cells (DCs) that inhibit CD8+ cytotoxic T cell activation.^[^ ^152^ ^]^ Ablating Tregs restored CD8+ T cell activity, suggesting that therapeutic targeting of Treg‐DC interactions may alleviate immunosuppressive activity in the tumor microenvironment.

**Figure 10:**
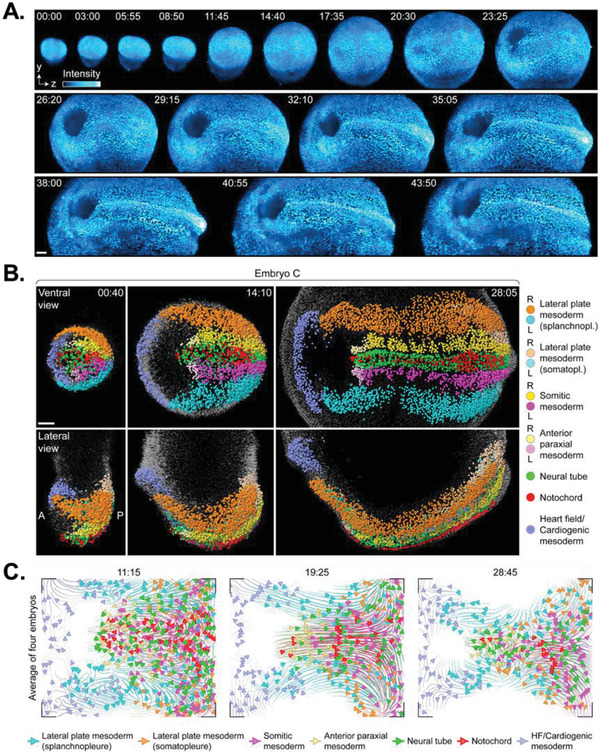
Light‐sheet imaging of live mouse embryos. A) Representative projections of H2B‐eGFP fluorescence in whole mouse embryos over 44 h of continuous imaging from early‐streak stage (E6.5) to somite (E8.5) stage. Scale bar is 100 µm. B) Dynamic cell fate map of a single embryo from mid/late streak stage to early somite stage at three time points. Scale bars is 200 µm. C) 2D representation of tissue movements from “averaged” mouse embryos at the mid‐bud, early head fold, and early somite stages. Reproduced with permission.^[^ ^158^ ^]^ Copyright 2018, Elsevier.

Another imaging modality, light‐sheet microscopy (LSM), optically sections live cells and tissues with “sheets” of light that enter the sample orthogonal to the objective and excite fluorophores that are imaged by wide‐field detection systems.^[^ ^153^ ^]^ Moving samples through the light sheet rapidly images the entire sample volume with minimal off‐target photodamage or perturbations to normal cellular behavior.^[^ ^154^ ^]^ This feature makes LSM particularly effective for studying photosensitive specimens ex vivo where surgical interventions are impractical such as developing embryos^[^ ^155^ ^]^ and 3D organoids.^[^ ^156^ ^]^ Moreover, repeatedly illuminating infinitesimally thin sections of a sample enables 3D subcellular dynamics in multicellular organisms to be captured with both spatial and temporal resolution, revealing the molecular consequences of cell–cell interactions throughout the sample volume.^[^ ^157^ ^]^ LSM was recently used to visualize and annotate the development of whole postimplantation mouse embryos with single‐cell resolution over a 48 h period^[^ ^158^ ^]^ (**Figure** **10**A). Tracking the migration and division of individual cells over time permitted the construction of a cell fate database that mapped the spatial origins of lineage plasticity (Figure 10B). Statistically combining cell fate data from multiple embryos produced a “composite” embryo from which averaged behaviors such as tissue morphogenesis could be statistically quantified with single‐cell resolution (Figure 10C). Thus, this work provides a dynamic reference for uncovering how the behaviors of individual cells (e.g., motility, proliferation, cell–cell interactions) evolve over time and space and contribute to the earliest stages of organogenesis.

## 4 Engineered Molecular Tools to Study Cell–Cell Communication

**Figure 11:**
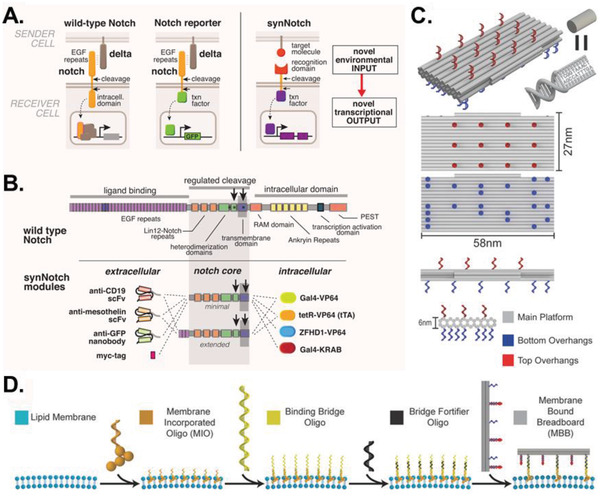
Engineering synthetic cell–cell communication pathways. A) Diagram of concepts underlying synNotch design. B) Intracellular and extracellular domains of the synNotch module can be swapped to engineer diverse ligand recognition sites and effector mechanisms, respectively. Reproduced with permission.^[^ ^168^ ^]^ Copyright 2016, Elsevier. C) Honeycomb lattices of double‐stranded DNA helices (gray tubes) can be conjugated on the top and bottom with molecular overhangs to form membrane bound breadboards (MBBs). D) Cell membranes studded with membrane incorporated oligos (MIOs) can be functionalized with MBBs through binding bridge oligos. The MBBs can in turn be modified to present user‐designed oligos. Reproduced with permission.^[^ ^174^ ^]^ Copyright 2017, Wiley‐VCH.

Augmenting how cells send and receive biological information can program cellular behavior,^[^ ^159^ ^]^ yield mechanistic insights into the biology of in vivo signaling processes,^[^ ^160^ ^]^ and produce innovative cell‐based therapies that target aberrant signaling networks associated with injury^[^ ^161^, ^162^ ^]^ and disease.^[^ ^163^, ^164^ ^]^ Genetic engineering strategies can achieve these insights by programming cells to produce synthetic cell–cell signaling molecules that interact with or replace native machinery. For example, the JAK‐STAT^[^ ^165^ ^]^ and Notch^[^ ^166^ ^]^ pathways are highly conserved cell–cell signaling networks that regulate immune cell function and numerous developmental programs, respectively. Since both pathways operate without intracellular signaling intermediates, they are exemplary platforms for designing communication networks that regulate transcriptional activity independently of endogenous processes. Recent studies have developed chimeric forms of JAK‐STAT cytokines (called synthekines)^[^ ^167^ ^]^ and Notch receptors (called synNotchs)^[^ ^168^ ^]^ that enable novel cell–cell communication mechanisms and trigger biologically distinct signaling programs upon activation with therapeutic applications (**Figure** **11**A,B). For example, synthekines can help realize the immunotherapeutic potential of endogenous cytokines (e.g., interleukin‐2, IL‐2)^[^ ^169^ ^]^ by reducing off‐target effects, while synNotchs have improved the specificity of engineered T cells called chimeric antigen receptor (CAR) T cells, which express modified T cell receptors that target tumors in adoptive cell therapy.^[^ ^170^, ^171^, ^172^ ^]^ Moreover, designing synthetic signaling systems that are sensitive to varying concentrations of user‐specified ligands and control a reporter gene could reveal context‐specific cell–cell communication in vivo in real time.^[^ ^173^ ^]^ Together, these approaches present synthetic biologists with valuable tools for inducing customized cell behaviors through user‐defined inputs.

Nongenetic techniques are also available to direct cell–cell signaling that transiently modify cell membrane proteins, making them more widely applicable to cell types like stem cells that are sensitive to genetic alterations.^[^ ^163^, ^174^, ^175^ ^]^ These approaches either modify native surface proteins or graft synthetic biomolecular structures onto cell surfaces to bestow non‐native functions. For example, self‐organizing DNA nanostructures, also known as DNA origami,^[^ ^176^ ^]^ were recently used to reversibly attach synthetic DNA scaffolds to the surfaces of adherent, suspension, and primary cell types on which numerous functions such as directed cell–cell contacts and fluorescence readouts could be programmed^[^ ^174^ ^]^ (Figure 11C,D). Integrating these nanostructures into cell membranes enabled precise control over homotypic and heterotypic adhesions, demonstrating the potential of DNA nanodevices to mimic complex biological processes and extend cellular functionality. Overall, these advances allow researchers to not only profile endogenous cell–cell communication mechanisms, but also design their own mechanisms that elucidate how complex cellular behaviors arise from cell–cell interactions.

## 5 Conclusions and Outlook

Mechanistic insights into the intricate cell–cell interaction networks that comprise multicellular organisms require innovative approaches. With conventional tools, researchers must choose between profiling a broad range of features with limited resolution or studying specific features with higher resolution, all within the same layer of biological information. Additionally, conventional strategies typically overlook the biological contexts of cell–cell signaling by reporting on bulk measurements that lack spatiotemporal and cellular resolution. Recent advances in microengineered and molecular tools have enabled the study of cell–cell interactions with unprecedented granularity, advancing our knowledge of fundamental cell biology and enabling the development of novel therapeutic strategies that address a diverse range of diseases such as cancer progression and aging.^[^ ^177^ ^]^

Microengineered platforms provide fine spatiotemporal control over cell–cell interactions to enable high‐throughput studies of how specific interactions modulate cell behavior in engineered microenvironments. 3D culture techniques and organs‐on‐chips have enabled researchers to reconstitute in vivo intercellular interactions in vitro with increasing fidelity, while single‐cell pairing and micropatterning tools resolve the heterogeneity among interacting cells. However, microfluidic strategies typically sacrifice multiparametric analyses in favor of cellular throughput, providing a limited toolbox with which to measure the consequences of cell–cell communication. For example, while microwell arrays are suitable for measuring the secretory output and behavior of individual cells in response to external stimuli, they are not ideal for studying cellular responses to dynamic soluble cues or monitoring the early stages of cell–cell contacts. Many microfluidic platforms rely on fluorescent labeling to identify and characterize interacting cells,^[^ ^178^ ^]^ which, while informative, lacks the advanced feature multiplexing capabilities seen in recent molecular profiling strategies. Thus, these platforms provide limited insights into the molecular mechanisms that govern cell–cell interactions. Moreover, since biological processes that depend on cell–cell contacts (e.g., differentiation and injury repair) occur over time scales ranging from hours to days, the ideal microengineered platform would arrange cells with fine spatiotemporal control and comprehensively profile their molecular states until these processes are resolved. We anticipate that advances in microengineered fabrication techniques will enable multiplexed cellular and molecular readouts that resolve the contributions of intrinsic and extrinsic cues to cell behaviors in the context of cell–cell interactions.

The continued development of spatial molecular profiling techniques will inevitably lead to substantial improvements in our understanding of how different layers of biological information interact with each other. These tools allow researchers to map not only the individual cell types that make up complex tissues, but also the diverse cell states that arise due to the molecular and cellular components in their immediate microenvironment. While most of these tools profile fixed tissues, advances in live‐cell imaging are making promising progress toward real‐time measurements. As strategies for multiplexed feature detection improve, commensurate advances in computational power will be required to process and interpret the results.^[^ ^179^, ^180^ ^]^ Multiplexed molecular validation techniques must also be developed for sequencing techniques that search for cell–cell interactions across entire tissues or organisms. For example, while techniques like ligand–receptor mapping can identify putative cell–cell interactions by the proportionate expression of ligand–receptor pairs, inferred results must be validated with more stringent molecular techniques that demonstrate the physical colocalization of each pair. Additionally, single‐cell sequencing and spatial molecular profiling expose cellular traits with such breadth and granularity that transient, latent cell states have been identified in response to various stimuli during homeostasis, tissue repair, and disease. These findings have sparked an ongoing debate regarding whether cell identities should be defined as discrete types or as a spectrum of transient cell states.^[^ ^181^, ^182^ ^]^ As our molecular understanding of cell–cell interactions deepens, we anticipate that traditional classifications of cell identity based on morphology and cell markers will be replaced with classifiers that depend on both biological and methodological factors. Molecular engineered tools like synNotch may play a central role in this effort by identifying the core molecular components that govern cell–cell interactions, and revealing which components are necessary and sufficient to shape cell fate with real‐time readouts. In the future, these tools may enable researchers to essentially reverse engineer in vivo signaling dynamics and reproduce them in vitro for further study.

Integrating the single‐cell manipulation capabilities of microengineered tools with the spatial omics‐level readouts of molecular engineered techniques will lead to new biological insights. A microfluidic device was recently developed to perform high‐throughput screens of chemotherapeutic drug efficacy in whole tumor sections through secretory immunophenotyping, revealing the extent to which the tumor microenvironment modulates drug responsiveness.^[^ ^183^ ^]^ Similarly, organs‐on‐chip systems are emerging that accommodate high‐throughput drug screening and sequencing methods. For example, a heart‐on‐a‐chip was constructed with a capacity to operate 35 replicates within a single chip and test the effect of Isoproterenol on cardiac contractility.^[^ ^184^ ^]^ More recently, patient‐derived cancer cells were cultured as 3D spheroids in barcoded nano‐wells that enabled paired, automated imaging of a 3D culture system with RNA‐seq, termed Pheno‐seq.^[^ ^185^ ^]^ Using the Pheno‐seq system, paired imaging and RNA‐seq data of 210 MCF10A spheroids and 95 patient‐derived colorectal cancer spheroids were acquired. Gene expression profiles varied considerably between spheroids that different in size. While these technologies incorporate both microengineered and molecular profiling strategies beyond fluorescence imaging, integrated omics‐level measurements have yet to be developed. We anticipate that future advances will allow cell–cell interaction networks in whole tissues to be perturbed with cellular resolution via microengineered strategies and the molecular consequences read out at the omics‐scale.

## Conflict of Interest

The authors declare no conflict of interest.
